# The efficacy of a leukotriene receptor antagonist in the treatment of human rectal aberrant crypt foci: a nonrandomized, open-label, controlled trial

**DOI:** 10.1186/s12885-020-07266-6

**Published:** 2020-08-17

**Authors:** Takuma Higurashi, Jun Arimoto, Keiichi Ashikari, Tomohiro Takatsu, Noboru Misawa, Tsutomu Yoshihara, Tetsuya Matsuura, Akiko Fuyuki, Hidenori Ohkubo, Atsushi Nakajima

**Affiliations:** Department of Gastroenterology and Hepatology, Yokohama City University School of Medicine, 3-9 Fukuura, Kanazawa, Yokohama, Kanagawa 236-0004 Japan

**Keywords:** Colorectal Cancer, Chemoprevention, Leukotriene receptor antagonist, Aberrant crypt foci

## Abstract

**Background:**

Leukotriene receptor antagonists (LTRAs) are broadly used for the management of allergic asthma and have recently been indicated to inhibit carcinogenesis and cancer cell growth. In colorectal cancer (CRC) chemoprevention studies, the occurrence of adenoma or CRC itself is generally set as the trial endpoint. Although the occurrence rate of CRC is the most confident endpoint, it is inappropriate for chemoprevention studies because CRC incidence rate is low in the general population and needed for long-term monitoring. Aberrant crypt foci (ACF), defined as lesions containing crypts that are larger in diameter and darker in methylene blue staining than normal crypts, are regarded to be a fine surrogate biomarker of CRC. Therefore, this prospective study was designed to explore the chemopreventive effect of LTRA on colonic ACF formation and the safety of the medicine in patients scheduled for a poly resection as a pilot trial leading the CRC chemoprevention trial.

**Methods:**

This study is a nonrandomized, open-label, controlled trial in patients with colorectal ACF and polyps scheduled for a polypectomy. Participants meet the inclusion criteria will be recruited, and the number of ACF in the rectum will be counted at the baseline colonoscopic examination. Next, the participants will be assigned to the LTRA or no treatment group. Participants in the LTRA group will continue 10 mg of oral montelukast for 8 weeks, and those in the no treatment group will be observed without the administration of any additional drugs. At the end of the 8-week LTRA intervention period, a polypectomy will be conducted to evaluate the changes in the number of ACF, and cell proliferation in the normal colorectal epithelium will be analyzed.

**Discussion:**

This will be the first study to investigate the effect of LTRAs on colorectal ACF formation in humans.

**Trial registration:**

This trial has been registered in the University Hospital Medical Information Network (UMIN) Clinical Trials Registry as UMIN000029926. Registered 10 November 2017.

## Background

Cancer is a one of the major health issues and a leading cause of death globally. The incidence, prevalence, and mortality rates of colorectal cancer (CRC) continue to rise all over the world [1, 2]. The majority of CRC cases are derived from adenomatous polyps [3], and their resection has been shown to reduce the risk of the future development of advanced adenomas and CRC [4, 5], thereby preventing CRC-related deaths [6]. However, patients with polyps (adenomatous polyps and/or hyperplastic polyps) represent a high-risk group for the occurrence of metachronous colorectal polyps and/or CRC [7]. Then, a paradigm is shifting from surveillance for the early detection of advance adenomas or CRCs and resection to novel tactics for prevention is required to reduce the mortality rate of this disease. A number of large-scale epidemiologic and/or clinical trials have assessed the prophylactic agents, including vitamin D, calcium, fiber and non-steroidal anti-inflammatory drugs (NSAIDs), such as aspirin and selective cyclooxygenase-2 (COX-2) inhibitors, in preventing against CRC development [8]. We previously reported that the NSAID sulindac suppressed the development of sporadic colorectal adenomas [9]. To this point, NSAIDS, particularly COX-2 inhibitors, have been proven to offer the greatest possibility for reducing CRC risk, either alone or in combination with other drugs [5], while it has been reported an elevated risk of serious cardiovascular events related with the administration of COX-2 inhibitors [10, 11]. In view of these adverse cardiovascular effects and the lack of efficacy of other drugs that initially looked promising, the development of novel agents that meet both safety and efficacy in preventing CRC is essential.

Leukotriene receptor antagonists (LTRAs), such as montelukast and zafirlukast, are commonly used for the treatment of allergic asthma [12, 13], and Tsai MJ et al. reported that LTRAs reduced cancer risk in a dose-dependent manner in asthma patients [14]. It was also reported that the cancer incidence rate was significantly lower in LTRA users than in non-LTRA users. (5.8 vs. 13.1 per 1000 patients/year). This means that apart from its role in asthma, LTRA has also been associated with carcinogenesis and tumor-mediated immunosuppression [15]. For example, the overexpression of cysteinyl leukotriene receptor 1 (CysLT_1_R) has been observed in CRC, and montelukast leads the apoptosis of CRC cancer cells [16, 17]. Previous in vivo studies have shown the chemopreventive effect of LTRAs [18, 19], but The chemopreventive effects of LTRA have not been studied in clinical practice. Therefore, we designed this study to ivestigate the chemopreventive effect of LTRAs in clinical practice. In CRC chemoprevention trials, the occurrence of adenomas or CRC itself is generally set as the trial endpoint. Though the occurrence of CRC is the most confident endpoint, it is not recommended for chemoprevention studies because CRC incidence rate is low in the general population and needed for long-term monitoring. Furthermore, there are ethical concerns about conducting long-term trials to determine whether a test agent is effective or not.

Aberrant crypt foci (ACF), defined as lesions containing crypts that are larger in diameter and darker in methylene blue staining than normal crypts [20–23], are regarded to be a fine surrogate biomarker of CRC [24]. Our group has previously reported that ACF is useful biomarker for CRC [25, 26] and study endpoint for a chemoprevention study [27, 28]. The advantages of chemoprevention studies with the number of colorectal ACF as the trial endpoint are that long-term observation is not needed to investigate the agent efficacy, and the number of ACF can be quantitatively estimated. Therefore, we set the ACF count as a good endpoint for this study. To the knowledge of us, this is the first clinical study investigating the use of LTRAs as chemopreventive agents against colorectal ACF in humans.

## Methods

### Study design and setting

This study was designed as a nonrandomized, open-label, controlled trial to be conducted in patients with colorectal ACF. It will be performed at the Department of Gastroenterology and Hepatology, Yokohama City University (YCU) Hospital, Japan. The coordinating office will be at the YCU Hospital, and patient registration will be conducted at the YCU center for novel and exploratory clinical trials (Y-NEXT) and data collection will be done using electronic data caputure.

### Ethical considerations and trial registration

The trial protocol complies with the Declaration of Helsinki [29] and the Ethical Guidelines for Clinical Research of the Ministry of Health, Labour, and Welfare, Japan [30]. Ethical approval of this trial was obtained from the Ethics committee of YCU Hospital on 30 May 2017. The study protocol and informed consent documents were approved by the YCU Hospital ethics committee. This trial has been approved in the Clinical Trial Act in Japan and registered in the Japan Registry of Clinical Trials (jRCT) as jRCTs031180094 and the University Hospital Medical Information Network (UMIN) Clinical Trials Registry as UMIN000029926. All study participants will submit a written study participation informed consent form.

### Participation criteria

We will recruit the patients with colorectal ACF and resectable polyps for this trial. The inclusion criteria are as follows: (1) patients with resectable polyps [adenoma, hyperplastic polyp, and sessile serrated adenoma/polyp (SSA/P)], (2) patients with more than five rectal ACF, and (3) submit written study participation informed consent form.

The exclusion criteria are as follows: (1) patients with lesions suitable for early removal, (2) a history of LTRA use within 2 months before study participation, (3) a history of malignant disease within 5 years before study participation, (4) a history of heart, renal, liver failure or liver cirrhosis, (5) a history of familial adenomatous polyposis, hereditary nonpolyposis CRC, or inflammatory bowel disease, (6) pregnancy or the possibility of pregnancy, (7) prohibitions of montelukast, (8) allergies to montelukast, (9) regular use of NSAIDs, metformin, and pioglitazone, and (10) participants considered as unsuitable for the study by the researchers.

### Intervention

All eligible participants will be assigned to the LTRA or no treatment group. Because this is an open-label trial, patients will be assigned to the no treatment group after the inclusion of patients in the LTRA group. Participants in the LTRA group will receive 10 mg of oral montelukast for 8 weeks, and those in the no treatment group will be observed without the administration of any additional drugs. At the end of the 8-week LTRA treatment period, a polypectomy will be performed to evaluate the changes in the number of ACF, and cell proliferation in the normal colorectal epithelium will be analyzed.

### Outcome measurements

The primary endpoint will be the change in the number of colorectal ACF after 8 weeks of treatment. A magnifying colonoscope (PCF-Q290AZI, HZ290; Olympus Co., Tokyo, Japan) will be used in all cases. Procedure preparation for the colonoscopy will begin 1 day before the procedure. Each participant will be informed to take a low-residue diet and 5 mg oral sodium picosulfate on the evening before the procedure. On the day of the procedure, each participant will be given 1500 ml polyethylene glycol (PEG). If the stools are not clear enough, an additional 500 ml PEG will be given to ensure adequate bowel cleansing. In most cases, conscious sedation with midazolam (3–5 mg) and pentazocine (7.5–15 mg) will be use at the start of the colonoscopy. Subcutaneous scopolamine or glucagon will be administered for colonic movements reduction. At the time of the first colonoscopy, the endoscopists will insert into the cecum, and the observe entire colorectum as the endoscope is pulled back. One colonic mucosal sample will be collected. The number of rectal ACF will be counted using a magnifying endoscope. At the end of the 8-week LTRA treatment period, the same endoscopists will perform the polypectomies and count the number of ACF. Endoscopists will record all procedures on a hard disk drive, and take photograph all ACF. The number of ACF in each participant will first be counted by the endoscopists during the procedure. To provide additional validation, the number of ACF will be recounted by three blinded expert endoscopists (AJ, HT, and AK) by observing the recorded hard disk drive. Cases that these evaluators deem colonoscopy to be inappropriate will be excluded from the final analysis.

The secondary outcomes will be as follows. (1) Drug safety: adverse events (AEs) will be graded according to the National Cancer Institute Common Toxicity Criteria for Adverse Events (NCI-CTCAE) version 4.0. All trial participants will be provided with a trial record for the daily dose of the trial agent and AEs. Participants who develop serious AE of grade 3 or higher will be discontinued at that time in the study. (2) The effects of LTRAs on cell proliferation in the rectal mucosa: one colonic mucosal sample will be collected from the same study patient by performing a biopsy at the time of the baseline colonoscopy and polypectomy. A biopsy will be obtained from all participants. Cell proliferation will be evaluated by Ki67 staining. Briefly, we will randomly select six crypts and count the number of Ki67-positive cells per crypt. In total, ~ 250 cells will be counted at a magnification of × 400 using a bright-field microscope. The results will be presented as the percentage of Ki67-positive cells. All participants will receive laboratory tests and a physical examination at the point of the baseline colonoscopy and polypectomy.

### Drug supply

Montelukast capsules will be purchased from Kyorin Corporation Ltd., Tokyo, Japan. Participants will be informed to take one tablet of the study drug every night before bed. Medication adherence will be monitored by counting the empty medication sheets returned by the participants at the time of their polypectomy. The participants will also be interviewed and monitored to confirm that they have not used any prohibited drugs (aspirin, metformin, and/or other NSAIDs). AEs will be monitored by the investigator and graded according to the NCI-CTCAE version 4.0. If serious AEs or less than 80% drug adherence are confirmed in a participant, this participant will be withdrawn from the trial.

### Sample size estimation and allocation

We previously reported the administration of 250 mg metformin per a day for 4 weeks reduced the number of ACF. In that trial, the mean number of ACF per patient decreased significantly from 8.8 ± 6.5 at baseline to 5.1 ± 5.0 at 4 weeks (*p* = 0.007) [28]. Although this study is exploratory research, and the accurate chemopreventive efficacy of montelukast is unknown, we assume that montelukast may have an effect that is equivalent to 60% of that observed for metformin on the reduction of ACF nubmer. Therefore, we estimate that the ACF number will change by about − 2 to − 3 on average. We determined that a sample size of 11–24 individuals in the LTRA group was needed to detect a significant reduction in the number of ACFs in the LTRA group using a paired t-test with a two-sided significance level of 5 and 80% power. Assuming some dropouts, we propose to recruit 30 participants in the LTRA group. To confirm that the number of ACF does not change during the study period, we propose to recruit 10 patients in the no treatment group after consecutively accumulating 30 patients in the LTRA group. Therefore, we propose to recruit 40 patients.

### Statistical analysis

The change in the number of ACF, which is the primary endpoint, will be compared before and after the 8-week study period between the LTRA and no treatment groups by the paired *t*-test. Drug safety will be assessed by the chi-square test, and the remaining results will be compared by the *t*-test or Mann–Whitney *U* test between the two groups. *P* < 0.05 will be defined as statistically significant. All statistical analyses will be conducted using JMP® 15 software (SAS Institute Inc., Cary, NC, USA).

### Trial steering committee and data monitoring committee

The Trial Steering Committee and Data Monitoring Committee will be located at the Y-NEXT. The Management Team will perform central monitoring of the trial status and data collection every month.

### Study flow

**Fig. 1:**
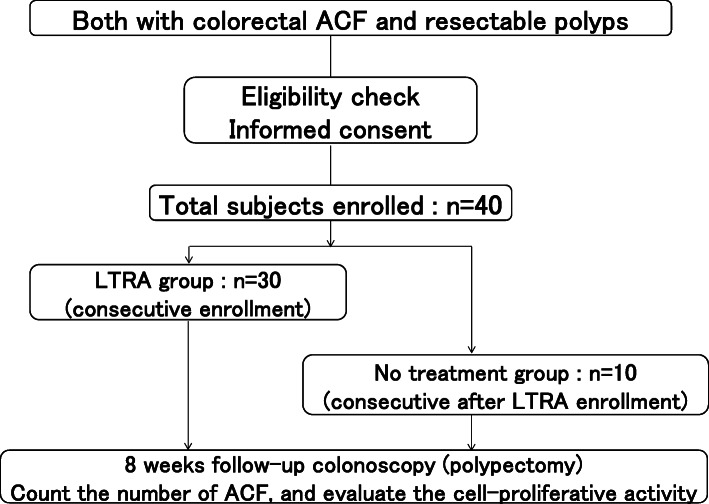
Study flow chart

A study flow is shown in Fig. 1.

## Discussion

To the knowledge of us, this is the first clinical trial proposed to investigate the efficacy of LTRAs on colorectal ACF formation. LTRAs are broadly used for the treatment of allergic asthma and rhinitis [12, 13], and LTRAs are reported to decrease the risk of cancer in asthma patients in a dose-dependent manner [14]. Previous basic research has reported that CysLT_1_R is overexpressed in CRC and that montelukast induces the apoptosis of CRC cells [16, 17]. CysLTs have recently been focused on as significant regulators of gut homeostasis, with endogenous CysLT production mediating the proliferation and survival of gut mucosal cells [31].

Recent evidence focuses on the effect of leukotriene C_4_ (LTC_4_) in accelerating oxidative DNA damage, if not adequately repaired, can contribute to increase mutation rates and genomic instability [32]. DNA damage and genomic instability are major drivers of carcinogenesis [33]. CysLTs also acts as leukocyte chemoattractants. In addition, CysLT_1_ mediates Th17 cell migration, the storage of which associates with the progression of inflammation-associated cancers [34]. Chronic inflammation is a risk factor for cancer initiation and progression, as observed in patients with inflammatory bowel disease [35]. Furthermore, leukotriene D_4_ (LTD_4_) antagonists suppress chronic inflammation in a rodent model of acute enteritis and this may be effective in preventing inflammation-associated CRC [36]. LTRAs are leukotriene pathway inhibitors, and thus they may have potential as chemotherapy and/or chemoprevention agents to reduce the effect of leukotrienes. Previous in vivo studies have elucidated the chemopreventive effect of leukotriene pathway inhibitors [18, 19] and showed the potential use of LTRAs for chemoprevention. Therefore, we will conduct this trial to explore the chemopreventive effect of LTRAs in clinical setting.

This trial may have some limitations as follow. First, ACF are believed to be a fine surrogate biomarker of CRC, though its biological significance in humans is still controversial [20]. In CRC chemoprevention studies typically set the occurrence of adenomas or the CRC itself as endpoint of the study. Though the occurrence of CRC is the most appropriate endpoint, it is inappropriate for chemoprevention studies because CRC incidence rate is low in the general population and needed for long-term monitoring. Our group has previously reported that ACF is useful biomarker for CRC and conducted a chemoprevention study for colorectal ACF [25, 26]. Therefore, we designed this study using the number of ACF as the primary endpoint to investigate the chemopreventive effect of LTRAs. Second, an intervention duration of 8 weeks may be too short to reliably detect differences between two groups. Since, our group reported in a previous trial that oral administration of metformin for 4 weeks reduced the number of colorectal ACF in humans [28]. An intervention period of 8 weeks should be enough to assess the changes in the number of ACF, if LTRAs have a chemopreventive effect.

Our group previously conducted a short-term chemoprevention study of metformin for colorectal ACF and reported the preventive effect of the agent on the formation of ACF. Then we conducted a one-year metformin chemoprevention study for colorectal polyps. We propose to repeat the same steps as in our metformin study for the chemoprevention study using montelukast.

If LTRAs were proved to have efficacy for CRC prevention, the impact would be significant. Therefore, we believe it will be very interesting to assess whether LTRAs inhibit the formation of human colorectal ACF.

## Data Availability

The datasets used and/or analyzed during the current study will be available from the corresponding author on reasonable request.

## Abbreviations

CRC: Colorectal cancer
NSAIDs: Non-steroidal anti-inflammatory drugs
COX-2: Cyclooxygenase-2
LTRAs: Leukotriene receptor antagonists
CysLT_1_R: Cysteinyl leukotriene receptor
ACF: Aberrant crypt foci
YCU: Yokohama City University
UMIN: University hospital Medical Information Network
SSA/P: Sessile serrated adenoma/polyp
NCI-CTCAE: National Cancer Institute Common Toxicity Criteria for Adverse Events
PEG: Polyethylene glycol
AEs: Adverse events
LTC_4_: Leukotriene C_4_
LTD_4_: Leukotriene D_4_
